# Risk factors for developing high-output ileostomy in CRC patients: a retrospective study

**DOI:** 10.1186/s12893-021-01288-y

**Published:** 2021-06-26

**Authors:** Dongxiao Bai, Lei Li, Zhiling Shen, Tianchen Huang, Qingbing Wang, Yanjun Wang, Yong Zhang, Zhipeng Guo, Kan Li, Jian an Xiao

**Affiliations:** Department of Surgery, Anyang Tumor Hospital, No. 1 Huanbin North Road, Anyang, 455000 Henan China

## Abstract

**Background:**

Anastomotic leakage is one of the most serious postoperative complications of rectal cancer. Prophylactic ileostomy has been widely used to reduce the risk and severity of complications of anastomotic leakage. However, prophylactic ileostomy itself has some complications, and ileostomy high output syndrome (HOS) is one of them. This study was performed to explore the risk factors of HOS in ileostomy.

**Methods:**

A total of 114 patients with HOS were screened out from 494 eligible ileostomy patients in the last 5 years. The relationship between HOS and the clinicopathological data was analyzed using the Chi-square test and Fisher’s exact probability. Multivariate analysis was performed by logistic regression.

**Results:**

The incidence of HOS was 23.07% in this study. Dehydration was the most common symptom of HOS (37.7%). There was no clear correlation between HOS occurrence with sex, age, gross typing, histological grade, tumor location, lymph node metastasis, and TNM stage (p > 0.05). The incidence of HOS was 14/18 in inflammatory bowel disease patients, 18/28 in diabetes mellitus patients, and 23/72 in neoadjuvant chemoradiotherapy patients, 13/17 in total colectomy and abdominal infection patients. Multivariate analysis showed that they are risk factors for HOS (p < 0.05).

**Conclusion:**

HOS occurred occasionally but rarely studied and lacks attention. Inflammatory bowel disease, diabetes mellitus, neoadjuvant radiotherapy chemotherapy, total colectomy and abdominal infection are the risk factors for HOS.

## Background

Among Chinese, colorectal cancer (CRC) is the fourth most common tumor in men and women. It accounts for 10.46% of all new cancer cases in men and 9.17% in women. Furthermore, the incidence of colorectal cancer is increasing [1]. Fortunately, more standardized and individualized treatments have reduced mortality and improved CRC patients’ quality of life. Radical surgery is still the primary means of treatment, but some patients with low rectal cancer have anastomotic fistula after surgery. Prophylactic ileostomy has been widely used to reduce the risk and severity of complications of anastomotic leakage [2, 3].

In general, ileostomy indications include colon injury, colorectal cancer, familial polyposis, ulcerative colitis, crohn’s disease, etc. For some advanced colorectal cancer patients with obstruction, it is necessary first to remove the intestinal obstruction with an ostomy, or an ileostomy is also feasible to carry out adjuvant therapy. For some middle and low rectal cancer and left colon cancer, edema, or others with high-risk factors of anastomotic fistula, it may be necessary to adopt preventive ileostomy to reduce the risk and complications of anastomotic leakage when resecting the lesion and one-stage anastomosis [4]. We generally agreed that preventive (prophylactic) ileostomy should be undergone on patients with the following situations. Patients underwent preoperative radiotherapy, especially those intestinal wall texture is brittle, with obvious congestion and edema; Low and ultra-low anastomosis, especially for those less than 3 cm. Poor proximal intestinal blood supply; Unsatisfactory anastomosis, large intestinal tension after anastomosis or positive intraoperative inflatable test; Age greater than 73 years old, poor cardiopulmonary function, malnutrition, anemia, and chronic persistent obstruction patient or long duration; Diabetes patients with poor blood glucose control, the long-term use of hormone.

However, prophylactic ileostomy itself has some complications, and ileostomy high output syndrome is one of them. The risk factors and predictors of high-output ileostomy have been reported in studies with a small sample size [5, 6]. We conducted a single-institution retrospective review of CRC patients who underwent ileostomy (both prophylactic and therapeutic) in our hospital, and the clinical and pathological data were analyzed to explore the risk factors of high-output ileostomy.

## Methods

The patients with colorectal cancer who underwent ileostomy for 5 consecutive years from March 2013 to March 2018 were collected in Anyang Tumor Hospital of Henan province, China. Patients enrolled were between 26 and 87 years old, with complete medical records, and were followed up either with ileostomy reversal or more than 1 year after ileostomy. HOS was defined as patients whose daily stoma output is more than 2000 ml and lasted more than 2 days, as a previous study reported [7]. The others were excluded from the group whose stoma discharge was not detailed and did not meet the follow-up requirements. One hundred fourteen patients (63 males and 51 females) with HOS were screened out from 494 eligible ileostomy patients in the last 5 years.

Among the 494 patients with enterostomy, 343 patients (68.6%) underwent preventive ileostomy during anterior resection (AR) or low anterior resection (LAR), 26 patients underwent ileostomy for bowel obstruction, 17 patients underwent a total colon resection. Emergency operations were performed on five patients. There were 1319 rectal operations cases during 2013–2018, and 343 patients underwent preventive ileostomy (accounting for 26.0%).

Early HOS (EHOS) was defined as HOS developed within 3 weeks after the operation, and late HOS (LHOS) was those over 3 weeks.

We collected clinical data using patient medical records, outpatient follow-ups, pathological stages, and complications like stoma discharge, electrolyte imbalance, dehydration, and disease treatments. Discharged patients were followed up to 1 year after ileostomy or ileostomy reversal.

SPSS17.0 statistical software was used for the statistical analysis; single-factor analysis was performed using the Chi-square test and Fisher’s exact probability method. The multifactor analysis was performed with logistic regression. p < 0.05 had statistical significance.

## Results

**Fig. 1:**
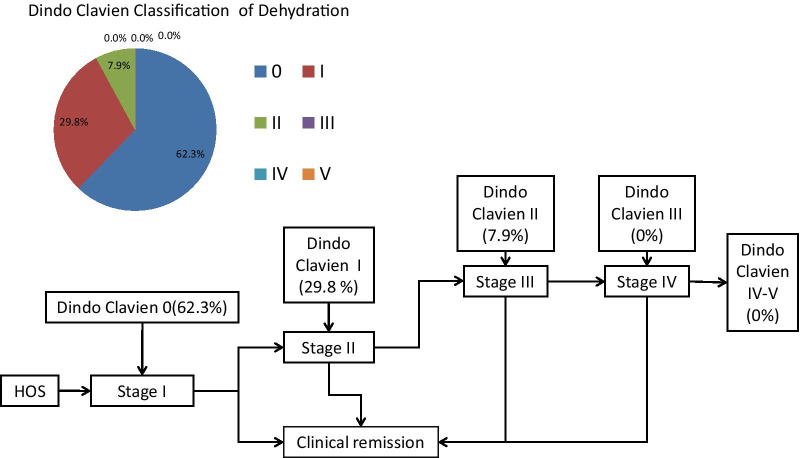
Management protocol and outcome of HOS patients. STAGE I: exclude potential causes. STAGE II: Initial management—reduce fluid and electrolyte losses. STAGE III: Ongoing HOS—optimise treatment with anti-secretory (somatostatin)/diarrheal (loperamide) medication. STAGE IV: Evaluate efficacy of additional treatment if HOS continues (Adapted from Baker et al. [7], Franklin Adaba et al. [17])

1. Common symptoms of HOS. Dehydration was the most common symptom of HOS (37.7%). Those patients need rehydrate with intravenous fluid or additional anti-secretory (somatostatin)/diarrheal (loperamide) medication treatment are 29.8% and 7.9% respectively (Fig. 1). Followed symptoms are electrolyte disturbance (28.1%), local dermatitis (21.9%), renal dysfunction (5.3%), and malnutrition (7.0%) in long-term HOS (Table 1).
2. Correlation between HOS and clinicopathological features of CRC. The results showed no clear correlation between the occurrence of HOS with gender, age, gross typing, histological grading, tumor location, lymph node metastasis, and TNM staging in patients with colorectal cancer (Table 2).
3. Correlation between HOS and preoperative treatments and concomitant diseases. These data showed that patients with inflammatory bowel disease, diabetes mellitus, and neoadjuvant chemoradiotherapy are associated with HOS occurrence (Table 3).
4. HOS and surgical-related indicators. These figures showed that total colectomy and postoperative abdominal infection are correlated with the occurrence of HOS but not with operation time, bleeding volume, application of diuretics, and laparoscopy (Table 4).
5. EHOS/LHOS and related pathological factors. The study demonstrated there is no significant difference between the occurrence of HOS and related pathological factors (inflammatory bowel disease, diabetes, mellitus, hypoproteinemia, neoadjuvant chemotherapy, anemia, etc.) in ileostomy patients (Table 5).
6. Multivariate analysis of related factors with HOS. The indicators related to the occurrence of HOS in univariate analysis were further analyzed by multivariate analysis. This study showed that preoperative situations including inflammatory bowel disease, diabetes mellitus and neoadjuvant chemoradiotherapy were risk factors for HOS (p < 0.05) (Table 6).

**Table 1:** Composition ratio of common symptoms of HOS

HOS	(n = 114)	Proportion (%)
Dehydration	43	37.7
Electrolyte disturbance	32	28.1
Hyponatremia	21	18.4
Hypochloritemia	18	15.8
Hypokalemia	14	12.3
Hypomagnesemia	4	3.5
Renal dysfunction	6	5.3
Malnutrition	8	7.0
Local dermatitis	25	21.9

**Table 2:** Relationship between HOS and clinical-pathological features

Clinicopathological	Cases	Indicators HOS n (%)	*P* value
Gender			0.741
Male	271	61 (22.5)	
Female	223	53 (23.8)	
Age			0.537
> 60 years old	193	44 (22.8)	
≤ 60 years old	291	70 (24.1)	
Gross type			0.726
Massive type	219	53 (24.2)	
Ulcerative type	162	38 (23.5)	
Infiltrating type	113	23 (20.4)	
Organizational grading			0.197
Highly differentiated	178	36 (20.2)	
Medium differentiation	191	42 (22.0)	
Poorly differentiated	125	36 (28.8)	
Tumor site			0.487
Rectum	351	79 (22.5)	
Colon	143	35 (24.5)	
Lymph node metastasis			0.487
Yes	307	74 (24.1)	
No	187	40 (21.4)	
TNM staging			0.934
Phase I	55	13 (23.6)	
Phase II	85	20 (23.5)	
Phase III	229	50 (21.8)	
Phase IV	125	31 (24.8)

**Table 3:** Univariate analysis of preoperative situations in HOS and non-HOS groups

Complications	Ileostomy n (%)		*P* value
	HOS (n = 114)	Non-HOS (n = 380)	
Inflammatory bowel disease	14 (12.3)	4 (1.1)	< 0.01*
Diabetes	18 (15.8)	10 (2.6)	< 0.01*
Mental disorder (tension)	5 (4.4)	15 (3.9)	0.84
Intestinal obstruction	6 (5.3)	21 (5.5)	0.91
Hypoproteinemia	11 (9.6)	42 (11.1)	0.96
Anemia	6 (5.3)	22 (5.8)	0.83
Neoadjuvant chemoradiotherapy	23 (20.2)	49 (12.9)	0.04*
Steroid hormones	4 (3.5)	6 (1.6)	0.18

^*^The difference was statistically significant

**Table 4:** Univariate analysis of surgical related indicators in HOS and non-HOS groups

Clinicopathological indicators	Ileostomy n (%)		*P* value
	HOS (n = 114)	Non-HOS (n = 380)	
Radical operation			< 0.01*
Radical resection of rectal cancer	67 (58.8)	250 (65.8)	
Radical resection of colon cancer	34 (29.8)	126 (33.2)	
Total colectomy	13 (11.4)	4 (1.1)	
Laparoscopic surgery			0.467
Yes	81 (71.1)	283 (74.5)	
No	33 (28.9)	97 (25.5)	
Operative time (h)			0.352
≥ 3	61 (53.5)	222 (58.4)	
< 3	53 (46.5)	158 (41.6)	
Surgical bleeding volume (ml)			0.514
≥ 300	38 (33.3)	115 (30.3)	
< 300	76 (66.7)	265 (69.7)	
Postoperative abdominal infection	8 (7.0)	5 (1.3)	0.000*
Postoperative application of Diuretics	3 (2.6)	4 (1.1)	0.204

^*^ The difference was statistically significant

**Table 5:** The relationship between EHOS, LHOS and related pathological factors

Clinicopathological factors	HOS (n = 114) n (%)		*P* value
	EHOS (n = 69)	LHOS (n = 45)	
Inflammatory bowel disease	10 (14.5)	4 (8.9)	0.279
Diabetes	11 (15.9)	7 (15.6)	0.956
Mental disorder (tension)	3 (4.3)	2 (4.4)	1.000
Hypoproteinemia	8 (11.6)	3 (6.7)	0.520
Anemia	4 (5.8)	2 (4.4)	1.000
Neoadjuvant chemoradiotherapy	11 (15.9)	12 (26.7)	0.163
Steroid hormones	3 (4.3)	1 (2.2)	0.483

**Table 6:** Logistic regression multivariate analysis

Clinicopathological factors	B	S.E	Wals	Sig	Exp (B)	95%CI
Inflammatory bowel disease	3.517	.636	30.555	.000	33.686	9.680–117.229
Diabetes	3.061	.508	36.244	.000	21.351	7.882–57.841
Neoadjuvant hemoradiotherapy	2.149	.425	25.533	.000	8.580	3.727–19.749
Total colectomy	1.191	.354	11.344	.000	3.291	1.646–6.584
Steroid hormones	3.470	.701	24.472	.000	32.146	8.133–127.060

## Discussion

HOS or high-output syndrome (basically the same) is rarely studied and lacks attention. There is no consensus on the definition of HOS [5, 7–10]. Dehydration, electrolyte disturbance (hyponatremia, hypochloremia, hypomagnesemia), renal failure, and malnutrition (late-stage) can occur in high-output ileostomy [5, 7, 8]. HOS increases the risk of readmission of CRC patients. Some scholars defined HOS as stoma output of more than 1500 ml per day for more than 2 consecutive days [8, 9], while the others advocate that HOS should be defined as the output of more than 2000 ml per day for more than 2 or 3 consecutive days [5, 7]. As the complications are more likely to occur when the daily stoma output exceeds 2000 ml, we adopt the latter standard in our study. HOS can be classified as early (< 3 weeks after initial ostomy surgery) or late HOS (3 weeks after surgery), and previous studies widely accept this.

In our cohort, the incidence of HOS (114/494) was 23.07%, higher than earlier reports (17%) [7]. Dehydration was the most common symptom of HOS (37.7%), followed by electrolyte disturbance (28.1%), local dermatitis (21.9%), renal dysfunction (5.3%), and malnutrition (7.0%) in long-term HOS. It was reported that the incidence of HOS is 26% in 262 patients with ileostomy during hospitalization, 30% of patients were re-admitted within 30 days after discharge, and 37% of patients with re-hospitalization were due to dehydration [11]. The readmission risk of ileostomy patients with inflammatory bowel disease was double that of other risk factors (OR 2.04) [12].

Our study found no clear correlation between the occurrence of HOS and gender, age, gross typing, histological grading, location of tumors, lymph node metastasis, and TNM staging in patients with colorectal cancer. Preoperative complications of inflammatory bowel disease, diabetes mellitus, and neoadjuvant chemoradiotherapy are risk factors for HOS. Inflammation caused by an abnormal reaction of the intestinal mucosal immune system act as an important role in the pathogenesis of inflammatory bowel disease and is also the leading cause of HOS. Diabetes mellitus occurred in patients with colorectal cancer because of the disorder of glucose metabolism and utilization. Examples such as inappropriate control, blood sugar, or intestinal movements may result in a large amount of liquid discharged from ileostomy [5]. We found that neoadjuvant concurrent radiotherapy and chemotherapy (CRT) is a risk factor for HOS (HOS). Radiation enteritis caused by neoadjuvant radiotherapy might be responsible for HOS in preoperative CRC patients [13]. As neoadjuvant chemoradiotherapy is mostly carried out simultaneously before an operation in our hospital, there is no stratified study of neoadjuvant chemotherapy, radiotherapy alone, and neoadjuvant concurrent radiotherapy and chemotherapy. It has been reported that preoperative radiotherapy alone, the distance between the tumor and anal margin, could affect the intestinal function of patients after operation, while chemotherapy alone has no significant effect on intestinal function after operation [14].

Among the related factors of operation, total colectomy and abdominal infection are the risk factors of HOS, but the operation time, bleeding volume, diuretic application, and laparoscopic operation is not. High stoma displacement in inflammatory bowel disease for most IBD patients was complicated with long-term diarrhea before surgery, and we believe that high stoma displacement is mainly related to the primary disease. Long-term oral administration of drugs (non-steroidal anti-inflammatory drugs) and hormone use may also be factors. Diabetes mellitus and total colectomy are high-risk factors for HOS, consistent with a previous study by Takeda and others.[5]. As reported, bile acid deficiency is one of the mechanisms of total colectomy caused by HOS. Total proctocolectomy prevents the reabsorption of bile acids absorbed by the ileocecum. As a result decrease in bile acid pools inhibits lipid absorption. Consequently, unabsorbed long-chain fatty acids are hydroxylated or desaturated by anaerobic intestinal bacteria, triggering the secretion of fluid and electrolytes, which may lead to the development of HOS [5]. Bile acid deficiency may also cause changes in the intestinal flora that increase intestinal drainage [15]. The loss of water absorption by the colon in patients undergoing total colectomy can also lead to high ileostomy output. Our study did not find that the use of steroids and diuretics after ileostomy increases the high output of ileostomy, but large doses of diuretics may increase the risk of dehydration.

The aims of management of patients with HOS are to: Provide nutrients, electrolyte, and water necessary to maintain health and growth, reduce the severity of intestinal failure, prevent and treat complications due to intestinal failure, achieve a good quality of life [16, 17]. Generally, half of the EHOS patients required no particular drug intervention until recuperation, while the other half required drug intervention [7]. For the treatment and management of HOS, patients should be managed and guided throughout the hospital and after discharge [18]. In addition to basic treatments (such as restricting fluid intake, rehydration, and correcting electrolyte disturbance), reducing the secretion of somatostatin and oral intake of loperamide effectively reduces discharge. Pieter-Jan Cuyle reported that 17% of patients with ileostomy had high excretion [19]. This complication would affect the implementation or completion of adjuvant therapy. Somatostatin analogues (ranitides, etc.) could reduce the excretion of ileostomy effectively. The routine dosage of loperamide is 20 mg twice daily by mouth, but the dosage can be increased if it is not effective. Alicia Mackowski reported that increasing the dosage of loperamide to 30 mg per day in individual patients can reduce the amount of stoma excretion, without observed abnormality in renal function [20]. Unfortunately, we cannot record the specific time of HOS patients’ return and only make a classification based on 3 weeks.

This paper is a retrospective study with some limitations. A prospective study should pay attention to the patients with high-risk factors HOS, strengthen the supervision and treatment after the operation and discharge for reducing the complications of stoma, and facilitate the smooth progress of comprehensive treatment and the readmission rate caused by HOS [21–24]. Furthermore, HOS generally increases the length of hospitalization, the cost of hospitalization, and the readmission rate, leading to clinical pathway variation, while details are not yet available. ICU transfer rate and follow-up survival were not included in the original data of this study.

We followed up with patients who had ileostomy reversal or 1 year after ileostomy by inpatient medical records, outpatient review, and telephone follow-up. These data mostly came from patients’ observation records in the hospital (including outpatient review) belonging to on-site data collection. Using remote video conferences to evaluate ileostomy output and taking early intervention measures to improve prognosis is a method worth exploring [25]. Multicenter, large sample randomized controlled studies of HOS should be conducted in the future.

## Conclusion

These results indicated that inflammatory bowel disease, diabetes mellitus, neoadjuvant chemoradiotherapy, total colectomy and abdominal infection are the risk factors for HOS. Such patients should be alert to the occurrence of HOS in perioperative period of CRC.

## Data Availability

The datasets used during the current study are available from the corresponding author on reasonable request.

## Abbreviations

CRC: Colorectal cancer
HOS: High-output stoma
EHOS: Early high-output stoma
LHOS: Late high-output stoma
